# Randomized double-blind placebo-controlled crossover trial with pyridostigmine in spinal muscular atrophy types 2–4

**DOI:** 10.1093/braincomms/fcac324

**Published:** 2022-12-09

**Authors:** Marloes Stam, Camiel A Wijngaarde, Bart Bartels, Fay-Lynn Asselman, Louise A M Otto, Laura E Habets, Ruben P A van Eijk, Bas M Middelkoop, H Stephan Goedee, Janke F de Groot, Kit C B Roes, Marja A G C Schoenmakers, Edward E S Nieuwenhuis, Inge Cuppen, Leonard H van den Berg, Renske I Wadman, W Ludo van der Pol

**Affiliations:** UMC Utrecht Brain Centre, Department of Neurology, University Medical Centre Utrecht, Utrecht University, 3584 CX Utrecht, the Netherlands; UMC Utrecht Brain Centre, Department of Neurology, University Medical Centre Utrecht, Utrecht University, 3584 CX Utrecht, the Netherlands; Child Development and Exercise Centre, Wilhelmina’s Children Hospital, University Medical Centre Utrecht, Utrecht University, 3584 EA Utrecht, the Netherlands; UMC Utrecht Brain Centre, Department of Neurology, University Medical Centre Utrecht, Utrecht University, 3584 CX Utrecht, the Netherlands; UMC Utrecht Brain Centre, Department of Neurology, University Medical Centre Utrecht, Utrecht University, 3584 CX Utrecht, the Netherlands; Child Development and Exercise Centre, Wilhelmina’s Children Hospital, University Medical Centre Utrecht, Utrecht University, 3584 EA Utrecht, the Netherlands; UMC Utrecht Brain Centre, Department of Neurology, University Medical Centre Utrecht, Utrecht University, 3584 CX Utrecht, the Netherlands; Biostatistics & Research Support, Julius Centre for Health Sciences and Primary Care, University Medical Centre Utrecht, 3584 CG Utrecht, the Netherlands; UMC Utrecht Brain Centre, Department of Neurology, University Medical Centre Utrecht, Utrecht University, 3584 CX Utrecht, the Netherlands; UMC Utrecht Brain Centre, Department of Neurology, University Medical Centre Utrecht, Utrecht University, 3584 CX Utrecht, the Netherlands; Child Development and Exercise Centre, Wilhelmina’s Children Hospital, University Medical Centre Utrecht, Utrecht University, 3584 EA Utrecht, the Netherlands; Knowledge Institute for Medical Specialists, Utrecht, the Netherlands; Biostatistics & Research Support, Julius Centre for Health Sciences and Primary Care, University Medical Centre Utrecht, 3584 CG Utrecht, the Netherlands; Department of Health Evidence, Section Biostatistics, Radboud University Medical Centre, Radboud University, 6525 EZ Nijmegen, the Netherlands; Child Development and Exercise Centre, Wilhelmina’s Children Hospital, University Medical Centre Utrecht, Utrecht University, 3584 EA Utrecht, the Netherlands; Department of Paediatric Gastroenterology, Wilhelmina’s Children Hospital, University Medical Centre Utrecht, Utrecht University, 3584 EA Utrecht, the Netherlands; UMC Utrecht Brain Centre, Department of Neurology and Child Neurology, Wilhelmina’s Children Hospital, University Medical Centre Utrecht, Utrecht University, 3584 EA Utrecht, the Netherlands; UMC Utrecht Brain Centre, Department of Neurology, University Medical Centre Utrecht, Utrecht University, 3584 CX Utrecht, the Netherlands; UMC Utrecht Brain Centre, Department of Neurology, University Medical Centre Utrecht, Utrecht University, 3584 CX Utrecht, the Netherlands; UMC Utrecht Brain Centre, Department of Neurology, University Medical Centre Utrecht, Utrecht University, 3584 CX Utrecht, the Netherlands

**Keywords:** spinal muscular atrophy, SMA, pyridostigmine, placebo, cross-over

## Abstract

Hereditary proximal spinal muscular atrophy causes weakness and increased fatigability of repetitive motor functions. The neuromuscular junction is anatomically and functionally abnormal in patients with spinal muscular atrophy. Pharmacological improvement of neuromuscular transmission may therefore represent a promising additional treatment strategy. We conducted a Phase II, monocentre, placebo-controlled, double-blind, cross-over trial with the acetylcholinesterase inhibitor pyridostigmine in treatment-naïve patients with spinal muscular atrophy types 2–4. We investigated the safety and efficacy of pyridostigmine on fatigability and motor function. Each participant received pyridostigmine and a placebo for 8 weeks, in random order. Primary outcomes were the repeated nine-hole peg test for fatigability and motor function measure. Secondary outcomes were patient-reported effects, endurance shuttle test combined scores and adverse events. We included 35 patients. For the repeated nine-hole peg test, the mean difference was 0.17 s/trial (95% confidence interval: −1.17–1.49; *P* = 0.8), favouring placebo, and for the motor function measure, 0.74% (95% confidence interval: 0.00–1.49; *P* = 0.05), favouring pyridostigmine. Around 74% of patients reported medium-to-large beneficial effects of pyridostigmine on fatigability, compared with 29.7% in the placebo arm. This was paralleled by a reduced dropout risk of 70% on the endurance shuttle test combined scores (hazard ratio: 0.30; 95% confidence interval: 0.15–0.58) under pyridostigmine. Adverse events, mostly mild and self-limiting, occurred more frequently under pyridostigmine. No serious adverse events related to the study medication were observed. Patients with spinal muscular atrophy tolerated pyridostigmine well. There were no significant differences in primary outcomes, but the self-reported reduction of fatigability and improved endurance shuttle test combined score performance suggest that pyridostigmine may be useful as an additional therapy to survival motor neuron-augmenting drugs.

*Trial registration number:* EudraCT: 2011–004369-34, NCT02941328

## Teaser

Stam *et al.* report on the placebo-controlled, double-blind, cross-over trial in 35 patients with spinal muscular atrophy types 2–4 to investigate the safety and efficacy of fatigability and motor function of the acetylcholinesterase inhibitor pyridostigmine. Pyridostigmine was well-tolerated, with suggestive evidence of reduced self-reported fatigability and improved performance on endurance tests.

## Graphical Abstract

**Graphical abstract:**
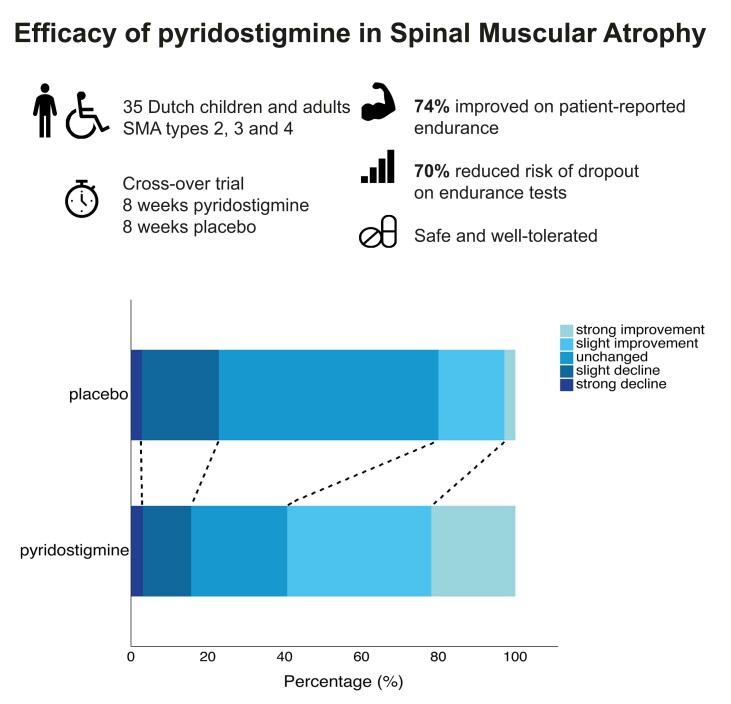

## Introduction

Hereditary proximal spinal muscular atrophy (SMA) is a severe neuromuscular disorder caused by the loss of function of the *survival motor neuron 1* (*SMN1*) gene.^1^ SMA is characterized by the degeneration of motor neurons in the anterior horns of the spinal cord, which results in progressive muscle atrophy and weakness.^2,3^ Recent reports show an additional abnormal development, maturation and function of the neuromuscular junction (NMJ).^4–6^ Although SMA is a monogenetic disorder, natural disease course shows a wide variety ranging from lethal infantile-onset SMA type 1 and chronic childhood variants types 2 and 3, to adult-onset type 4.^7^  *SMN* gene*-*modulating or -replacement therapies have become available in an increasing number of countries as of 2016. These therapies have been shown to improve survival and gross motor development in infants with SMA type 1 and motor function in young children and adults with SMA types 2 and 3.^8–15^

The main clinical feature of SMA is progressive muscle weakness, but patients also often complain of limited endurance of their remaining motor function while performing repetitive physical activities, such as using cutlery, brushing their teeth, writing and walking. This lack of endurance, or increased ‘fatigability’, is not only secondary to weakness, but is an additional dimension of motor impairment in patients with SMA.^16,17^ The disturbed development, maturation and functionality of the NMJ in SMA mouse models and patients with SMA types 1, 2 and 3 suggest that NMJ dysfunction may contribute to increased fatigability.^4–6,18–20^ Rare studies on treatments targeting NMJ abnormalities in SMA mouse models show beneficial effects on neuromuscular transmission, lifespan, muscle strength and motor function of agents aimed at voltage-gated calcium and potassium channels. However, the effects on fatigability have not been examined.^21^ Pyridostigmine is a widely used treatment for disorders affecting NMJ transmission, in particular myasthenia gravis. It enhances NMJ transmission by inhibiting the enzymatic breakdown of acetylcholine and consequently increasing its availability in the synaptic cleft.^22^ We, therefore, investigated the effect and efficacy of pyridostigmine on fatigability and motor function in patients with SMA types 2, 3 and 4.

## Material and methods

### Standard protocol approvals, registrations and patient consents

We conducted an investigator-initiated, Phase II, monocentre, placebo-controlled, double-blind cross-over trial at the Netherlands SMA Centre at the University Medical Centre Utrecht, a tertiary referral centre for patients with SMA in the Netherlands. The Medical Ethics Committee of the University Medical Centre Utrecht (14-536) and the Central Committee on Research Involving Human Subjects approved the study (NL3804804114). An external independent party (Julius Clinical, Zeist and The Netherlands) monitored the trial for accuracy, integrity and safety. All patients and parents or legal representatives, in case patients were <18 years old, gave written informed consent prior to study participation. We assigned a random study ID after inclusion to assure confidentiality. We conducted the study according to the principles of the Declaration of Helsinki (World Medical Association General Assembly 2013, Fortaleza, Brazil) and in accordance with the Medical Research Involving Human Subjects Act. This study is registered at the EU Clinical Trials Register (EudraCT number: 2011-004369-34, registration date: 29 August 2011, www.clinicaltrialsregister.eu) and ClinicalTrials.gov (NCT02941328, registration date: 16 September 2016). We enrolled the first patient in this study on 24 November 2015.

The reporting of this study conforms to the Consolidated Standards of Reporting Trials checklist (Supplementary Table 1).

### Patients

We published the trial design and procedures previously, including detailed inclusion- and exclusion criteria.^23^ In short, included patients were 12 years of age or older and had a genetically confirmed diagnosis of *SMN1-*related SMA types 2, 3 or 4. SMA types were defined as follows: disease onset >6 but <18 months of age and (being) able to sit unassisted but never able to walk unassisted for SMA type 2; disease onset >18 months, but <30 years of age and being able to walk unassisted at some point in life for SMA type 3; and disease onset >30 years of age and being able to walk unassisted at some point in life for SMA type 4. SMA type 3 was further subdivided into SMA types 3a and 3b based on age at onset before or after 3 years of age, respectively.^3^ All patients had to meet the predetermined minimum or maximum scores on the motor function measure (MFM) and the repeated nine-hole peg test (R9HPT).^24,25^ Absolute exclusion criteria were the use of drugs that alter NMJ function (e.g. salbutamol), a known concomitant disorder of the NMJ, or contra-indications for the use of pyridostigmine. None of the participants were treated with *SMN2-*splicing modifying drugs (i.e. nusinersen or risdiplam) because these were not reimbursed in the Netherlands during the course of this trial.

*SMN1* (HGNC:11117; OMIM600354) and *SMN2* (HGNC:11118; OMIM601627) copy number status was determined using SALSA multiplex ligation-dependent probe amplification kit P021 (version B1). All multiplex ligation-dependent probe amplification reactions were carried out according to the manufacturer’s protocol (www.mlpa.com; www.mrcholland.com).

### Treatment, randomization and study procedures

Trial design with 8-week periods was based on the following assumptions: (i) enough time for treatment dosing, adjustment to treatment and evaluation of effects during 8 weeks and (ii) no effects of natural disease progression by comparing two-treatment periods during 17 weeks.^2,26^

If eligible, patients were randomized in a permuted four-block design by an independent pharmacist who was not part of the study team, to receive either pyridostigmine or visually identical placebo tablets during the first 8-week treatment period. Patients crossed over to the other treatment for another 8-week period after a one-week washout period. Investigators and patients were blinded for treatment allocation.

Pyridostigmine is an orally active, reversible acetylcholinesterase inhibitor that increases extracellular acetylcholine levels in the NMJ by impairing the breakdown by acetylcholinesterase. Maximum plasma concentration is reached within 1–1.5 h, and half-life elimination occurs within 3–4 h in case of normal renal function.^27^ Bioavailability is 10–20% because of poor absorption in the gastrointestinal tract. Pyridostigmine does not cross the blood–brain barrier and therefore central effects are limited.^28^ Studies on the pharmacokinetics of pyridostigmine and its hydrolysis and liver enzymatic metabolism suggest that the resulting metabolites have little biological action.^27^

Patients were administered the study medication four times daily because of the short half-life of pyridostigmine, leading to an effect duration of ∼4–6 h. We used a dose-escalation period in the course of the first week of each treatment period to minimize side effects. Patients started on pyridostigmine 2 mg/kg/day, increased to 4 mg/kg/day and were eventually dosed at the final target of 6 mg/kg/day. If side effects after a dosage increase were not acceptable for the patient, the treatment period was continued with the highest tolerated dose. There were four study visits after the screening visit: one at the beginning and one at the end of each 8-week treatment period. During study visits, patients performed all clinical assessments in identical order, with a resting period in between assessments. We standardized study drug intake to 1 h before assessments.

Adverse events (AEs) after study participation were assessed by a telephone call after 1 week, or longer if potential adverse drug effects persisted.

The randomization code was revealed to the investigators after the database was closed in a two-step approach to reduce the risk of bias: first, the two study drugs were named ‘A’ and ‘B’ to perform the main analysis, followed by further unblinding.

### Outcomes

We published a detailed description of all outcome measures previously.^23^ A short description of the primary and most relevant secondary outcome measures is reported below.

Primary outcome measures were the R9HPT and MFM. The R9HPT is a fatigability test in which patients have to perform five consecutive rounds of the 9HPT as fast as possible.^24,29^ The MFM is a 32-item scale developed to measure the distal, proximal and axial motor function of patients with neuromuscular diseases, including SMA.^25^

As secondary outcome measures, we used patient-reported perceived treatment effect (global perceived effect on a rating scale of six: (1) completely recovered; (2) much improved; (3) slightly improved; (4) no change; (5) slightly worse; (6) much worse),^30^ the ‘risk of dropout’ and ‘time until dropout’ on the endurance shuttle test combined score (ESTCS), and AEs. The ESTCS has been validated to capture fatigability across the severity spectrum of SMA.^31^ Each patient performed one of three endurance shuttle tests (ESTs) that best matched his/her physical capacities. Patients with motor function of the hand and forearm performed the endurance shuttle nine-hole peg test, patients with anti-gravity motor function of at least one arm performed the endurance shuttle box and block test and ambulatory patients performed a modified version of the endurance shuttle walk test. All these tests mimic activities of daily living and all have the same construct, thus allowing combined analysis of both non-ambulant and ambulant patients with SMA types 2–4.^16,31,32^ As outlined previously, patients performed the EST at a constant, predetermined, individualized speed of 75% of their maximum ability for 20 minutes.^16,23,31,32^ If a test was not completed, we recorded the ‘time until dropout’ as the outcome for increased fatigability, i.e. the duration at which patients were unable to continue the EST.

### Statistical analysis

As reported previously, we calculated that we needed to include 40 patients in this trial to have enough power to detect meaningful changes in primary outcome measures.^14^ We aimed to recruit 45 participants with SMA types 2–4 based on two power calculations that we performed based on the cross-over design using pilot data on repeated measures of the total score of the MFM test (unpublished data). First, we calculated the within-participant standard deviation (SD), and next the SD of the difference between subsequent measurements of the participants. Calculation 1: If a total of 40 participants would enter this two-treatment cross-over study, the probability is 80% that the study would detect a treatment difference at a two-sided 0.05 significance level if the true difference between treatments is 1.093 units. This is based on the assumption that the within-participant SD of the response variable is 1.7 units. Calculation 2: If a total of 40 participants would enter this two-treatment cross-over study, the probability is 80% that the study would detect a treatment difference at a two-sided 0.05 significance level, if the true difference between treatments is 1.409 units. This is based on the assumption that the SD of the difference in the response variables is 3.1 units. Both calculations show similar results in terms of the detectable difference based on 80% power, a two-sided significance level of 0.05 and 40 participants in total in the trial. The five additional participants were scheduled to be recruited to compensate for potential dropouts. The total number of included participants we wanted to include was therefore 45.

We performed all efficacy analyses according to the intention-to-treat principle and included data from all patients that had at least one efficacy measurement (i.e. the efficacy population). We assessed primary outcomes individually, not co-primary, for statistical significance. We used linear mixed effect (LME) models to account for the intra-individual clustering of observations. For the MFM, we used an LME with fixed effects for the treatment period (1 or 2) and treatment (pyridostigmine or placebo). The random part was modelled with a random intercept per individual and an unstructured covariance matrix. For the R9HPT, we used a similar approach, with the addition of rounds (integers 1 to 5) as a fixed effect and a random slope of rounds per individual. We evaluated treatment response in interaction with rounds (i.e. does the duration of the 9HPT change differently over rounds under Treatments A versus B?). For all models, we tested for a period-treatment interaction to assess potential carryover effects. We used the likelihood ratio test to compare models; 95% CIs were based on the profile likelihood. We analysed the patient-reported perceived treatment effect with an LME similar to the MFM model. We analysed ‘time until dropout’ on the ESTCS using a Cox proportional hazards analysis with a Gaussian frailty term per subject; Kaplan–Meier estimates were presented to visualize the effect. Safety analyses were carried out based on the treatment received (i.e. safety population). We categorized AEs according to the Common Terminology Criteria for AEs and we summarized them in frequency tables, including severity ratings and likelihood of association with study medication.^33^ We used R (v3.6.0 for Windows with RStudio v1.2.1335) for all statistical analyses. The LMEs were fitted using the lmer function of lme4 (v1.1-21).^34^

We considered the results statistically significant when alpha was <0.05. Due to the exploratory nature of this Phase II study, results were not adjusted for multiple testing.

## Results

### Patients

Between November 2015 and August 2017, we screened 47 patients for eligibility and randomized 37 patients for trial participation. Prior to the start of using study medication, two patients discontinued trial participation, due to concomitant medical issues. Patient characteristics are summarized in Table 1.

**Table 1:** Baseline characteristics

		Pyridostigmine–placebo group (*n* = 17)	Placebo–pyridostigmine group (*n* = 18)
SMA type, *n* (%)	2	10 (59)	5 (28)
	3a	5 (29)	9 (50)
	3b	2 (12)	3 (17)
	4	0	1 (6)
Male gender, *n* (%)		8 (47)	5 (28)
Age in years, mean (SD; range)		34 (12; 13–53)	38 (14; 13–59)
Disease duration in years, mean (SD; range)		33 (12; 11–51)	34 (14; 11–59)
Ambulant, *n* (%)		2 (12)	2 (11)
MFM score, median (range)		37.5 (15.6–75)	36.5 (20.8–76)
*SMN2* copies, *n* (%)	2	1 (6)^a^	0 (0)
	3	7 (41)	8 (44)
	4	9 (53)	10 (56)

n = number; SD = standard deviation; MFM = motor function measure; SMN2 = survival motor neuron 2.

^a^ patient with SMA Type 2 and *SMN2* c.859G > C mutation.^35^

There were more male patients (eight versus five), more patients with SMA type 2 (10 versus 5) and less with SMA type 3a (five versus nine) in the group that started with pyridostigmine compared with the group that started with a placebo. Other characteristics did not differ between groups at baseline.

**Figure 1:**
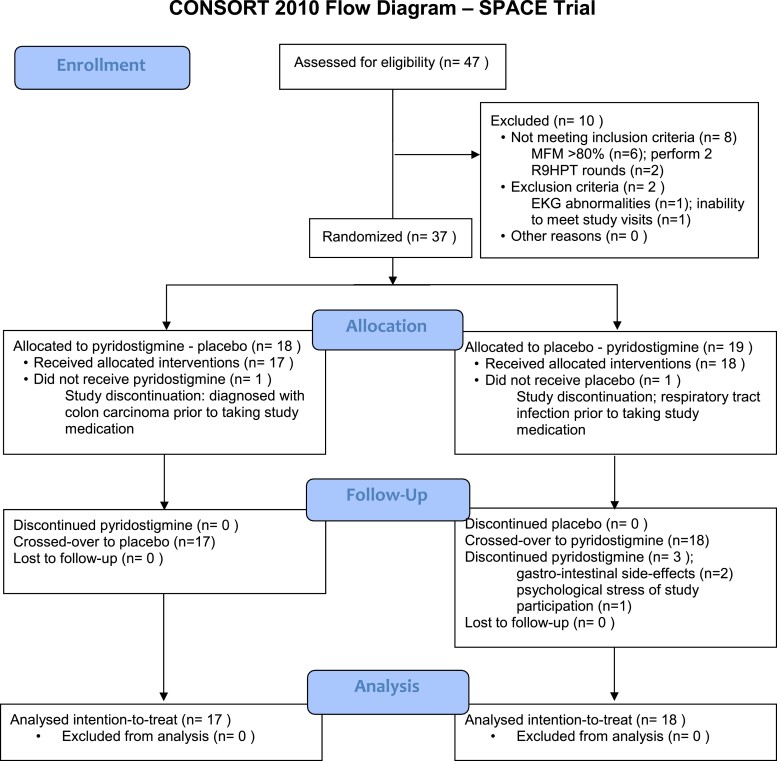
Patient flow diagram.

After the washout period, all 35 patients started the second period and 32 patients completed all study visits (Fig. 1). An intention-to-treat analysis was done on all 35 patients who started treatment. The maximum study medication dose had to be reduced to the best tolerated dose in nine patients due to side effects.

### Efficacy

Results are summarized in Table 2.

**Table 2:** Summary of outcome measures

			Placebo mean (95% CI)	Pyridostigmine mean (95% CI)	Mean difference (95% CI)	*P*-value
Primary outcomes	R9HPT, s/trial		0.70 (−0.42–1.81)	0.86 (−0.25–1.98)	0.17 (−1.17–1.49)	0.81
	MFM Total score		41.60 (35.95–47.26)	42.35 (36.69–48.00)	0.74 (0–1.49)	0.050
Secondary outcomes	ECTS^a^	Perceived treatment effect; odds of favourable effect under pyridostigmine, OR (95% CI)	—	6.9 (2.2–24.0)	—	
		Risk of dropout under pyridostigmine^a^, HR (95% CI)	—	0.30 (0.15–0.58)	—	
		Time until dropout, mean difference (95% CI) (pyridostigmine—placebo)	—	154 (48–216)	—	

CI = confidence interval; HR = hazard ratio; OR = odds ratio.

^a^ All 35 patients are included in the ESTCS. The 16 patients performed the ESNHPT, 15 patients performed the ESBBT and 4 patients performed the ESWT.

#### Primary outcomes

The time needed to complete one round during the R9HPT increased at a mean rate of 0.70 s/trial (95% CI: -0.42–1.81) under placebo and 0.86 s/trial (95% CI: −0.25–1.98) under pyridostigmine, resulting in a mean difference of 0.17 s/trial (95% CI: −1.17 to 1.49; *P* = 0.81), favouring placebo. There was a treatment-independent learning effect for the R9HPT, as the mean time to complete the test decreased by 2.89 s (95% CI: 0.98–4.81) between the first and second efficacy assessments.

The average MFM score was 41.6% (95% CI: 35.95–47.26) under placebo and 42.4% (95% CI: 36.69–48.00) under pyridostigmine, resulting in a mean difference of 0.74% (95% CI: 0.00–1.49; *P* = 0.050), favouring pyridostigmine. Results were similar in both intention-to-treat and per-protocol analyses.

#### Secondary outcomes

**Figure 2:**
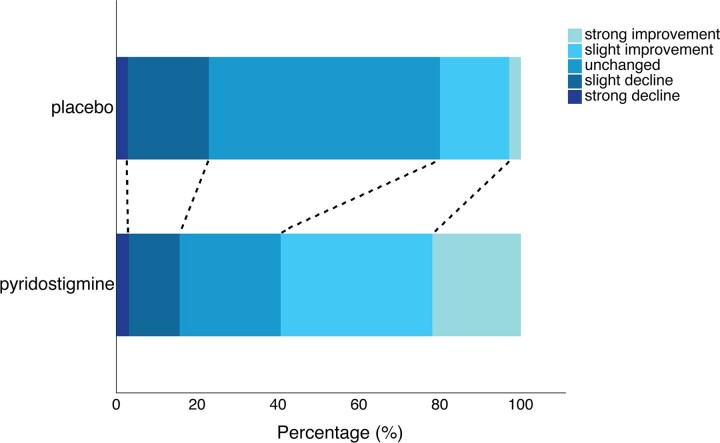
**Patient-reported perceived treatment effect on fatigability.** Patient-reported change in endurance at the end of each treatment period.^30^ Rating was done based on the following scale: (1) completely recovered; (2) much improved; (3) slightly improved; (4) no change; (5) slightly worse; (6) much worse. The odds of a favourable effect (i.e. a slight or strong improvement of fatigability) were higher under pyridostigmine compared with placebo (LME: OR 6.9, 95% CI: 2.2–24.0).

**Figure 3:**
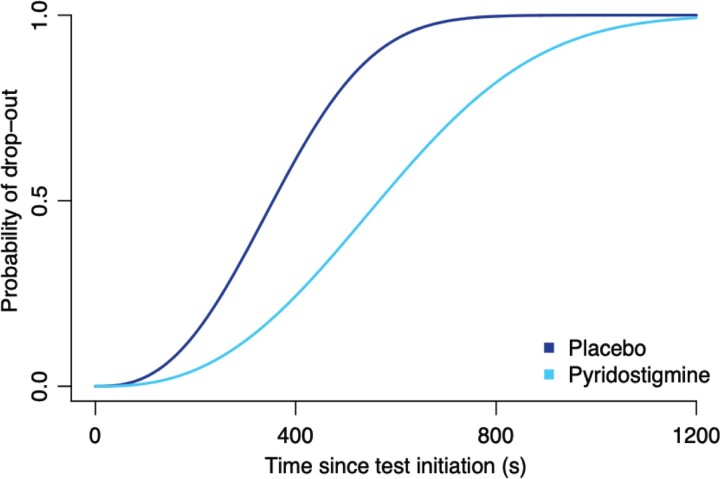
**Probability of test endurance on the ESTCS.** The probability of test endurance on the ESTCS visualized using Kaplan–Meier estimates; the interpretation is similar to that of a conventional survival curve. The number at risk table indicates the number of patients who have not dropped out. A Cox proportional hazard model with frailty terms was used to account for the within-patient dependence. The adjusted mean difference in ‘time until dropout’ was 154 s (95% CI: 48–216), favouring pyridostigmine.

Patients reported a beneficial effect of treatment with pyridostigmine on fatigability (74.4%, compared with 29.7% under placebo; Fig. 2). The odds of a favourable effect (i.e. a slight or strong improvement of fatigability) were higher under pyridostigmine compared with placebo [odds ratio (OR): 6.9, 95% CI: 2.2–24.0]. Pyridostigmine reduced the risk of dropout during the 20-min ESTCS by 70% compared with placebo (hazard ratio: 0.30; 95% CI: 0.15–0.58; Fig. 3). The adjusted mean difference in ‘time until dropout’ was 154 s (95% CI: 48–216), favouring pyridostigmine.

### Safety

We summarized AEs in Table 3. A detailed description of all AEs is available (Supplementary Table 2).

**Table 3:** Adverse events

	All	Placebo	Pyridostigmine	Follow-up
AEs (*n*)				
Cardiac disorders	2	0	2	0
Eye disorders	9	1	8	0
General disorders and administration site conditions	9	6	3	0
Infections and infestations	3	0	3	0
Metabolism and nutrition disorders	1	1	0	0
Psychiatric disorders	2	1	1	0
Reproductive system and breast disorders	2	1	1	0
Skin and subcutaneous tissue disorders	10	1	9	0
Vascular disorders	2	0	2	0
Gastrointestinal disorders	78	19	59	0
Musculoskeletal and connective tissue disorders	24	3	21	0
Nervous system disorders	14	8	6	0
Other	1	1	0	0
Renal and urinary disorders	17	4	13	0
Respiratory, thoracic and mediastinal disorders	20	13	6	1
Seriousness of event (*n*)				
Serious	5	2	2	1
Intermediate	18	4	14	0
Mild	171	53	118	0

Overall, AEs were more prevalent under pyridostigmine compared with placebo. Patients reported most AEs as mild, self-limiting and acceptable. Gastrointestinal complaints were the most reported AEs. Other prevalent related side effects were increased salivation and blurry sight. Participants reported muscle cramps and pain after study visits. All four serious adverse events (SAEs) were considered to be unrelated to study procedures or medication. The two SAEs occurred during the pyridostigmine treatment period. One patient underwent surgery following a diagnosis of colon carcinoma after study inclusion but prior to the start of study medication. Another patient was admitted to the hospital because of head trauma. The one patient with an extensive medical history of upper gastrointestinal tract bleeding was admitted to the hospital twice due to upper gastrointestinal tract bleeding; both episodes occurred during placebo use. Finally, one patient developed a severe respiratory tract infection several weeks after study completion. There were no signs of infection during the initial one-week follow-up period.

## Discussion

In this Phase II study, we found no statistically significant effect of pyridostigmine on the selected primary outcome measures, i.e. R9HPT performance and total MFM score, in patients with SMA types 2–4. Pyridostigmine was well-tolerated, with suggestive evidence that pyridostigmine may reduce fatigability, as shown by a reduction in self-reported fatigability in combination with the 70% reduced ‘dropout risk’ on the ESTCS. A larger trial is needed to confirm these findings, preferably with pyridostigmine both as monotherapy and as add-on therapy to SMN-augmenting drugs and with the recently validated ESTs as the primary outcome.^16,31,32^

Changes in motor function have been used as a primary outcome measure in most clinical trials for SMA.^8–15^ We used the MFM, a validated outcome measure for patients with SMA aged 6 years and older that has been used previously in a Phase II trial.^25,36,37^ The small difference in MFM scores between the treatment arms favoured pyridostigmine, but did not reach the prespecified level of statistical significance. We cannot exclude that we missed clinically meaningful changes in this trial, due to the relatively small sample size and short treatment periods. Reported improvements in motor function of SMN-augmenting therapies in young children and adults were observed after longer treatment periods.^8–15^

NMJ disorders are characterized by fatigability, e.g. decreasing performance of repetitive tasks. Fatigability is the most likely dimension of motor function to improve under pyridostigmine treatment since it is the first-line drug for NMJ disorders.^21,37^ At the same time, it is important to stress that motor function scales such as the MFM have not been designed to detect changes in the fatigability of remaining motor function. Therefore, we included fatigability tests that can be uniquely used across the spectrum of SMA severity. We used a combination of the R9HPT and the recently validated ESTs.^23^ Since the ESTs had not been validated when we designed this trial, we included them as secondary outcomes. An advantage of the ESTs is that, in contrast to the R9HPT, they are tailored to the individual’s remaining motor function and do not have a ceiling effect for patients with more extensive motor functions. We observed the ceiling effect of the R9HPT in patients with SMA type 3 in our previous study and it is therefore likely that this is also reflected in the outcomes of this trial.^24^ The ESTCS uniquely allows for combining results of patients with different levels of motor function, including both ambulatory and non-ambulatory patients, into a single analysis.^16,31,32^ This approach revealed a potential beneficial effect of pyridostigmine on fatigability (Fig. 3). The reduced fatigability under pyridostigmine as measured by the EST is convincingly supported by the patient-reported reduction in fatigability (Fig. 2). It is therefore important to confirm these findings in a future study. Since it is not certain whether SMN-augmenting drugs such as nusinersen, risdiplam and onasemnogene abeparvovec improve NMJ function in patients with SMA, pyridostigmine may provide additional benefits by specifically reducing symptoms of fatigability.

We hypothesized that pyridostigmine could improve fatigability by ameliorating the cholinergic pathway of SMA-related NMJ dysfunction. Additional putative effects of pyridostigmine have been suggested and include non-specific biological effects of pyridostigmine bromide on the cholinergic anti-inflammatory pathway (e.g. by inhibiting tumour necrosis factor-alpha)^38–42^ and balancing autonomic function.^39,43–48^ However, it is unknown if these potential putative effects are of any means in patients with SMA treated with pyridostigmine. This trial was not designed to study the effects on the NMJ itself and we cannot exclude other mechanisms of action. Although the suitability to evaluate treatment effect is unclear, nerve conduction studies with repetitive nerve stimulation to document the presence of decrement were part of the study protocol.^23^ A large number of missing values caused by the combination of low compound muscle action potential amplitudes and insufficient data quality (e.g. due to technical difficulties because of contractures) unfortunately precluded meaningful analysis. The data are included in the Supplementary Table 3.

From the reduction of muscle fatigability in other NMJ disorders, we infer that enhancement of neuromuscular transmission at the NMJ by pyridostigmine is the most likely mechanism for the improved performance of the ESTs.^22,49^ Targeting the NMJ may therefore be an important additional SMA treatment strategy. To the best of our knowledge, therapeutic interventions to ameliorate fatigability in patients with SMA have only been the subject of a few clinical trials. For example, beta2-adrenoreceptor agonist salbutamol and albuterol could improve NMJ function in patients with SMA,^50,51^ in addition to its proposed effects on muscle and SMN protein production.^52^ In a previous, small, placebo-controlled trial all participants with SMA reported improvements of perceived, subjective fatigability under salbutamol. These changes in fatigability were, however, not quantified.^53^ A recent pilot study with amifampridine, a voltage-dependent potassium channel blocker with pre- and postsynaptic NMJ effects, showed improvement of muscle strength, but not on timed tests including the 6-min walk test. Other validated fatigability tests were not included in the study protocol.^54^

Genetic *SMN*-modulating or replacement therapies (i.e. nusinersen, risdiplam and onasemnogene abeparvovec) improve survival in patients with SMA type 1 and motor function in patients with SMA types 1–3, but their effects on fatigability are unclear.^8–15^ A *post hoc* analysis of fourteen patients with SMA types 2 and 3 suggested a possible beneficial effect of nusinersen on fatigability.^55^ Additional, larger studies are still needed to assess the effects of genetic therapies on fatigability and NMJ function.

In our trial, none of the participants were treated with SMN*-*augmenting therapies, as these were not yet reimbursed during the duration of this trial. On a global level, many patients, especially those in lower-income countries, still do not have access to the costly *SMN2-*splicing modification therapies. Furthermore, genetic therapies do not provide a complete cure for SMA. Patients with significant motor disability at treatment initiation, benefit much less in terms of motor function gains.^14,15,56^ Therefore, pyridostigmine or similar alternatives may be of additional therapeutic value for patients with SMA. We think it would be particularly interesting to evaluate the possible additional effects of SMN-augmenting therapies in future clinical trials.

Pyridostigmine proved to be safe and well-tolerated. The vast majority of encountered side effects are well-known side effects of pyridostigmine (Supplementary File 1), and were mostly mild and self-limiting or resolved with dose reduction in 11 of 12 cases. Since we used a standardized dose in this study, it is possible that individual tailoring of the dose could result in an improved balance between efficacy and side effects. Dropouts as a result of cholinergic side effects were limited to two patients and occurred only in the pyridostigmine treatment period after placebo treatment. We therefore think the effects of unblinding and known treatment allocation by cholinergic side effects were limited.

Although pyridostigmine was well-tolerated in our selected patient cohort, we do not recommend pyridostigmine in patients with severe swallowing problems or severe respiratory insufficiency. Muscuranic side effects, including increased salivation, might result in (micro) aspiration and this will not outweigh the potential benefits of pyridostigmine.

A limitation of this trial is the sample size, which falls short of the original design. The two patients were unable to start the treatment, and three dropped out. The reason for dropout was the occurrence of cholinergic side effects in two out of three. Reduction of dosage (per-protocol) was needed in 11 out of 35 (30%) patients. Cholinergic side effects potentially have unblinded patients, but the dropout rate was the same in both study arms, either starting with a placebo or pyridostigmine period. Our study was therefore underpowered to detect relatively small and possibly clinically relevant changes in the primary outcomes and meaningful subgroup analyses could not be performed. We invited all patients with SMA types 2, 3 and 4 listed in the Dutch National SMA registry, which included over 300 patients at the start of this trial, to participate to decrease the risk of selection bias.^57^ We included patients with SMA types 2, 3 and 4, but our selection criteria excluded patients at the most severe ends of the spectrum. We used a cross-over design, in which patients act as their own controls, thereby reducing variance despite including different SMA types. Unfortunately, some patients were reluctant to participate in a medication trial and not all patients screened for eligibility met in the inclusion- and exclusion criteria (Fig. 1). We extended the enrolment period twice and after consultation with the SMA patient organization, we decided to finalize the study and analyse results with fewer participants than we had hoped to include. Even though we were unable to identify a significant difference in primary outcomes, the self-reported and EST-documented improvements suggest that pyridostigmine could reduce fatigability from repetitive tasks in patients with SMA.

This is the first and only placebo-controlled trial investigating the effect of pyridostigmine in treatment-naïve SMA patients. It is likely that, due to the current therapeutic landscape for SMA, it will not be possible to again recruit a large number of untreated patients. Future studies to further establish pyridostigmine efficacy will therefore need to be designed to include various SMN-augmenting therapies, and primarily evaluate its add-on effect to SMN-augmenting therapies. The primary clinical interest in such a trial should be endurance performance on repetitive motor tasks rather than the motor function itself, based on the most likely clinical and biological effect of pyridostigmine.

## Supplementary Material

fcac324_Supplementary_Data

## Data Availability

Fully anonymized data generated and analysed in this study are available from the corresponding author on reasonable request.

## Abbrevations

AE =: adverse event
EKG =: electrocardiogram
ESBBT =: endurance shuttle box and block test
ESNHPT =: endurance shuttle nine-hole peg test
EST =: endurance shuttle test
ESTCS =: endurance shuttle test combined scores
ESWT =: endurance shuttle walk test
MFM =: motor function measure
MLPA =: multiplex ligation-dependent probe amplification
NMJ =: neuromuscular junction
R9HPT =: repeated nine-hole peg test
SAE =: serious adverse events
SMA =: spinal muscular atrophy
SMN =: survival motor neuron
