# Supplementary material for: Randomized double-blind placebo-controlled crossover trial with pyridostigmine in spinal muscular atrophy types 2–4
Source: Brain Commun. 2022 Dec 9;5(1):fcac324. doi: 10.1093/braincomms/fcac324 (PMC9825780; doi:10.1093/braincomms/fcac324)
Supplement: fcac324_Supplementary_Data [file fcac324_supplementary_data.pdf]

Supplemental Data

Supplementary Table 1. CONSORT Checklist

CONSORT 2010 checklist of information to include when reporting a randomised trial*

Section/Topic	Item No	Checklist item	Reported on page No
**Title and abstract**			
	1a	Identification as a randomised trial in the title	1
	1b	Structured summary of trial design, methods, results, and conclusions (for specific guidance see CONSORT for abstracts)	1
**Introduction**			
Background and objectives	2a	Scientific background and explanation of rationale	4
	2b	Specific objectives or hypotheses	4
**Methods**			
Trial design	3a	Description of trial design (such as parallel, factorial) including allocation ratio	5-7
	3b	Important changes to methods after trial commencement (such as eligibility criteria), with reasons	6
Participants	4a	Eligibility criteria for participants	6
	4b	Settings and locations where the data were collected	5
Interventions	5	The interventions for each group with sufficient details to allow replication, including how and when they were actually administered	5-8
Outcomes	6a	Completely defined pre-specified primary and secondary outcome measures, including how and when they were assessed	5-8
	6b	Any changes to trial outcomes after the trial commenced, with reasons	5-8, 18
Sample size	7a	How sample size was determined	8
	7b	When applicable, explanation of any interim analyses and stopping guidelines	NA
Randomisation:			
Sequence generation	8a	Method used to generate the random allocation sequence	5-8
	8b	Type of randomisation; details of any restriction (such as blocking and block size)	6-7
Allocation concealment mechanism	9	Mechanism used to implement the random allocation sequence (such as sequentially numbered containers), describing any steps taken to conceal the sequence until interventions were assigned	6-7
Implementation	10	Who generated the random allocation sequence, who enrolled participants, and who assigned participants to interventions	6-7
Blinding	11a	If done, who was blinded after assignment to interventions (for example, participants, care providers, those assessing outcomes) and how	6-7
	11b	If relevant, description of the similarity of interventions	NA
Statistical methods	12a	Statistical methods used to compare groups for primary and secondary outcomes	8
	12b	Methods for additional analyses, such as subgroup analyses and adjusted analyses	8
**Results**			

Participant flow (a diagram is strongly recommended)	13a	For each group, the numbers of participants who were randomly assigned, received intended treatment, and were analysed for the primary outcome	9-15
	13b	For each group, losses and exclusions after randomisation, together with reasons	9-15
Recruitment	14a	Dates defining the periods of recruitment and follow-up	9-15
	14b	Why the trial ended or was stopped	9-15
Baseline data	15	A table showing baseline demographic and clinical characteristics for each group	Table 1
Numbers analysed	16	For each group, number of participants (denominator) included in each analysis and whether the analysis was by original assigned groups	9-15. suppl data
Outcomes and estimation	17a	For each primary and secondary outcome, results for each group, and the estimated effect size and its precision (such as 95% confidence interval)	9-15
	17b	For binary outcomes, presentation of both absolute and relative effect sizes is recommended	9-15
Ancillary analyses	18	Results of any other analyses performed, including subgroup analyses and adjusted analyses, distinguishing pre-specified from exploratory	NA
Harms	19	All important harms or unintended effects in each group (for specific guidance see CONSORT for harms)	9-15, suppl data
**Discussion**			
Limitations	20	Trial limitations, addressing sources of potential bias, imprecision, and, if relevant, multiplicity of analyses	15-19
Generalisability	21	Generalisability (external validity, applicability) of the trial findings	15-19
Interpretation	22	Interpretation consistent with results, balancing benefits and harms, and considering other relevant evidence	15-19
**Other information**			
Registration	23	Registration number and name of trial registry	4&5
Protocol	24	Where the full trial protocol can be accessed, if available	Suppl data
Funding	25	Sources of funding and other support (such as supply of drugs), role of funders	20

*We strongly recommend reading this statement in conjunction with the CONSORT 2010 Explanation and Elaboration for important clarifications on all the items. If relevant, we also recommend reading CONSORT extensions for cluster randomised trials, non-inferiority and equivalence trials, non-pharmacological treatments, herbal interventions, and pragmatic trials. Additional extensions are forthcoming: for those and for up to date references relevant to this checklist, see www.consort-statement.org.

Supplementary Table 2. Adverse Events

	Placebo						Treatment					
System Organ Class	patient no	description	severity	related to studymedication	study medication actions	outcome	patient no	description	severity	related to studymedication	study medication actions	outcome
Cardiac disorders							1	palpitaions	mild	unlikely	none	recovered
							25	extrasystole	mild	unlikely	none	ongoing
Eye disorders	21	teary eyes	mild	unlikely	none	recovered	1	teary eyes	mild	likely	none	recovered
							8	irritaion of the eye	mild	possibly	none	recovered
							14	blurry sight	intermediate	likely	reduction	recovered
							18	blurry sight	mild	likely	none	recovered
							25	teary eyes	mild	likely	none	unknown
							30	short events of blurry sight	mild	probably	none	recovered
							36	blurry sight	mild	likely	reduction	ongoing
							37	teary eyes in the morning	mild	possibly	none	recovered
Gastrointestinal disorders	1	stomach growling, nausea, diarrhoea	mild	possibly	none	ongoing	1	hypersalivation	mild	likely	none	recovered
	3	nausea	mild	possibly	none	recovered	1	stomach growling	mild	probably	reduction	ongoing
	3	thinner stool	mild	possibly	none	recovered	1	stomach ache	mild	probably	none	unknown
	5	stomach complaints	mild	possibly	none	recovered	1	hypersalivation and increased sweating	mild	likely	discontinuation	recovered
	5	diarrhoea	mild	possibly	none	recovered	2	stomach ache, increasing with palpation	mild	unlikely	none	ongoing
	5	obstipation	mild	unrelated	none	recovered	2	colon carcionoma (+ surgery)	serious	unrelated	discontinuation	recovered
	5	stomach ache	mild	possibly	none	recovered	3	stomach complaints	mild	probably	none	recovered
	5	bloated feeling	mild	possibly	none	recovered	3	diarrhoea	mild	likely	none	recovered
	10	increased stool frequency	mild	possibly	none	recovered	5	increased stool frequency	mild	likely	reduction	recovered
	12	stomach bleeding	serious	unrelated	temporarily pauzed	recovered	5	diarrhoea	mild	likely	reduction	recovered
	12	stomach ache + vomiting	serious	possibly	temporarily pauzed	recovered	5	stomach cramps	mild	probably	reduction	recovered
	13	diarrhoea	mild	possibly	none	ongoing	5	hypersalivation	mild	likely	none	recovered
	18	thinner stool	mild	possibly	none	recovered	5	stomach ache	mild	probably	none	recovered
	21	hypersalivation	mild	unlikely	reduction	unknown	5	obstipation	intermediate	unlikely	temporarily pauzed	recovered
	21	stomach ache	mild	possibly	reduction	recovered	6	stomach ache	mild	probably	none	recovered
	21	obstipation	mild	unrelated	none	recovered	7	hypersalivation	mild	likely	none	recovered
	35	stomach ache	mild	possibly	none	recovered	7	increased gut activity	mild	likely	none	recovered
	39	nausea	mild	possibly	none	recovered	8	stomach ache, nausea	mild	probably	none	recovered
	39	nausea + vomiting	mild	possibly	none	recovered	8	increased stool frequency	mild	likely	none	recovered
							8	thinner stool	mild	likely	none	recovered
							10	stomach cramps	mild	probably	none	recovered
							10	nausea and vomiting	mild	probably	none	recovered
							11	increased stool frequency	mild	likely	none	unknown
							12	stomach bleeding	intermediate	unrelated	none	recovered
							14	diarrhoea	mild	likely	none	recovered
							15	stomach ache	mild	probably	reduction	recovered
							15	diarrhoea	mild	likely	reduction	recovered
							15	hypersalivation	mild	likely	reduction	recovered
							16	hypersalivation	mild	likely	none	recovered
							16	diarrhoea	mild	likely	none	recovered
							17	thinner stool + increased frequency	mild	likely	none	recovered
							17	stomach ache	mild	probably	none	recovered
							17	hypersalivation	mild	likely	none	recovered
							18	hypersalivation	mild	likely	none	recovered
							18	diarrhoea	mild	likely	none	recovered
							20	stomach ache	intermediate	probably	reduction	recovered
							20	increased stool frequency	intermediate	likely	reduction	recovered
							21	nausea	mild	probably	reduction	recovered
							21	vomiting	mild	probably	temporarily pauzed	recovered
							21	stomach cramps	mild	probably	none	recovered
							22	stomach cramps + nausea	mild	possibly	none	recovered
							25	increased stool frequency	mild	likely	none	unknown
							27	nausea	mild	probably	reduction	recovered
							27	stomach ache	mild	probably	none	recovered
							29	oesophageal spasma	mild	probably	none	recovered
							29	stomach growling	mild	probably	none	recovered
							30	increased stool frequency + stomach growling	mild	likely	none	recovered
							31	stomach ache	mild	probably	none	ongoing
							31	thinner stool	mild	likely	none	recovered
							33	hypersalivation	mild	likely	none	recovered
							34	bloated feeling	mild	probably	none	recovered
							34	nausea and vomiting	mild	probably	none	recovered
							35	stomach ache	intermediate	probably	temporarily pauzed	recovered
							35	stomach ache	intermediate	probably	reduction	recovered
							35	hypersalivation	mild	likely	none	recovered
							36	stomach ache	intermediate	probably	reduction	recovered
							36	stomach ache	mild	probably	none	recovered
							37	thinner stool	mild	likely	none	recovered
							39	hypersalivation	mild	likely	none	recovered

**General disorders and administration site conditions**	3	feverish	mild	possibly	none	recovered	8	feverish	mild	possibly	none	recovered
	13	tiredness	mild	possibly	none	ongoing	18	feverish	mild	possibly	none	unknown
	25	unwell, tired, general weakness	mild	unrelated	none	recovered	32	extreme tiredness	mild	possibly	reduction	recovered
	29	tiredness (after horseriding)	mild	unrelated	none	recovered						
	37	cold	mild	unlikely	none	recovered						
	37	less energy	mild	unlikely	none	ongoing						
**Infections and infestations**							5	fever	mild	unlikely	none	recovered
							23	fungal infection	mild	unrelated	none	recovered
							37	vaginal bacterial infection	mild	unrelated	none	recovered
**Metabolism and nutrition disorders**	21	hypoglycemia	intermediate	unrelated	none	recovered						
**Musculoskeletal and connective tissue disorders**	3	stiffness, cramps, tired hand muscles	mild	unlikely	none	recovered	3	short episodes of light cramps in left hamd and foot	mild	possibly	none	recovered
	11	increase of pre-existent shoulder complaints	mild	unrelated	none	ongoing	4	cramp of neck muscles	mild	likely	none	recovered
	18	muscle pain	mild	unrelated	none	recovered	4	muscle cramps	mild	likely	reduction	Hersteld
							5	cramp of neck and jaw muscles	mild	likely	reduction	recovered
							5	cramps pectoral muscles	mild	likely	none	recovered
							5	trembling of muscles	mild	probably	none	recovered
							5	cramp in hand muscles	mild	likely	none	recovered
							11	muscle pain	mild	probably	none	recovered
							11	muscle spasms of right hand	mild	likely	none	recovered
							11	contusion of the foot/ankle	mild	unrelated	temporarily pauzed	recovered
							16	pain in left ankle after trauma	mild	unrelated	none	recovered
							18	trembling leg	mild	probably	none	recovered
							20	stiffness and cramps of hand muscles	mild	likely	reduction	recovered
							21	muscle cramps upper leg and gluteal muscles	mild	likely	none	recovered
							25	muscle cramps + light trembling of muscles	mild	likely	none	ongoing
							27	back pain	mild	unrelated	none	ongoing
							29	muscle spasms lower legs and face	mild	likely	none	recovered
							30	muscle spasms arms and legs	mild	likely	none	recovered
							31	muscle pain	mild	probably	none	recovered
							31	muscle spasms arms	mild	likely	none	ongoing
							32	increased muscle weakness	intermediate	unlikely	none	recovered
**Nervous system disorders**	3	dizziness	mild	unlikely	none	recovered	2	dizziness (end of the day - seeing black spots)	mild	unlikely	none	ongoing
	3	headache	mild	unlikely	none	recovered	4	vertigo	mild	possibly	reduction	recovered
	5	headache	mild	unlikely	none	recovered	5	tingling sensation at left side of the head	mild	unlikely	none	ongoing
	5	slight dizziness	mild	unlikely	none	recovered	10	headache	mild	unlikely	none	recovered
	14	headache	mild	unlikely	none	recovered	18	headache	mild	unlikely	none	recovered
	28	headache	mild	unlikely	none	recovered	39	concussion (fall off stairs while playing/running)	serious	unrelated	none	recovered
	33	headache	mild	unlikely	none	recovered						
	39	headache	mild	unlikely	none	recovered						
**Other**	16	globus sensation	mild	possibly	reduction	recovered						
**Psychiatric disorders**	1	emotional lability	intermediate	unrelated	none	ongoing	1	nervous, anxiety	mild	unlikely	discontinuation	unknown
**Renal and urinary disorders**	1	increased urination frequency	mild	unlikely	none	ongoing	2	increased urination frequency	mild	likely	none	ongoing
	10	increased urination frequency	mild	unlikely	none	recovered	4	increased urination frequency	mild	likely	none	ongoing
	31	urinary tract infection	mild	unrelated	none	recovered	5	increased urination frequency	mild	likely	reduction	recovered
	36	kidneystones with pyelonephritis	intermediate	unlikely	temporarily pauzed	recovered	8	increased urination frequency	mild	likely	none	recovered
							10	increased urination frequency	mild	likely	none	recovered
							12	pyelonephritis	intermediate	unrelated	none	recovered
							17	urinary urgency	mild	likely	none	recovered
							21	urinary tract infection	mild	unrelated	temporarily pauzed	recovered
							27	possible urinary tract infection	mild	unrelated	none	recovered
							31	urinary tract infection	intermediate	unrelated	none	recovered
							32	urinary tract infection	mild	unrelated	none	recovered
							36	increased urination frequency	mild	likely	reduction	recovered
							37	increased urination frequency	mild	likely	none	unknown
**Reproductive system and breast disorders**	5	late period (+1 week)	mild	unlikely	none	recovered	22	feeling unwell twice during period	mild	unrelated	none	recovered
**Respiratory, thoracic and mediastinal disorders**	3	symtomps of a cold	mild	unrelated	none	recovered	5	viral upper respiratory tract infection	mild	unlikely	none	recovered
	5	sore throat	mild	unrelated	none	recovered	8	symptoms of a cold	intermediate	unlikely	none	recovered
	5	upper respiratory tract infection	mild	unrelated	none	recovered	13	upper respiratory tract infection	mild	unlikely	none	recovered
	14	upper respiratory tract infection	mild	unrelated	none	recovered	15	coughing up mucus	intermediate	probably	reduction	recovered
	16	upper respiratory tract infection	mild	unrelated	none	recovered	18	changed sense of smell	mild	unrelated	none	ongoing
	17	cough	mild	unlikely	none	unknown	33	upper respiratory tract infection	mild	unlikely	none	recovered
	21	upper respiratory tract infection	mild	unrelated	none	recovered						
	23	tightness of the chest	mild	unrelated	none	recovered						
	23	upper respiratory tract infection	mild	unrelated	none	recovered						
	29	respiratory tract infection	intermediate	unrelated	temporarily pauzed	recovered						
	36	upper respiratory tract infection	mild	unrelated	reduction	recovered						
	37	upper respiratory tract infection	mild	unrelated	none	ongoing						
	38	upper respiratory tract infection	mild	unrelated	none	recovered						
	***** during follow-up - several weeks after study completion:***											
	8	pneumonia (ICU admittance)	serious	unrelated	n.a. (weeks after study completion)	recovered						

**Skin and subcutaneous tissue disorders**	36	sweating	mild	unlikely	none	recovered	4	increased sweating	mild	likely	none	ongoing
							10	sweating	mild	likely	none	recovered
							10	sweating	mild	likely	none	recovered
							10	sweating	mild	likely	none	recovered
							15	sweating	intermediate	likely	reduction	recovered
							17	sweating	mild	likely	none	recovered
							18	excessive sweating	mild	likely	none	recovered
							18	skin rash	mild	possibly	none	recovered
							37	increased sweating	mild	likely	none	recovered
**Vascular disorders**							27	painful legs and edema	mild	unrelated	none	ongoing
							37	more often edema in feet	mild	possibly	none	recovered

Supplementary Table 3. Results from conduction studies with repetitive nerve stimulation (RNS)

We performed nerve conduction studies with repetitive nerve stimulation (NCS-RNS) at each study visit.
Four different muscles are tested, assessing four different nerves: i.e. facial nerve: musculus nasalis; accessory nerve: musculus trapezius; median nerve: musculus flexor carpi radialis; and ulnar nerve: musculus abductor digiti minimi. Muscles were investigated for supramaximal CMAP recording and 3Hz repetitive stimulation (train of 10) in rest and after 60 seconds of maximal voluntary muscle activation. [1]
Pathological decrement is present when the 5th CMAP on RNS is ≤10% of the first CMAP.

In the table below we summarized the percentage change of the 5th CMAP compared to the first CMAP for all four nerves investigated at all four visits. Values of ≤10 mean that pathological decrement is present.

Subject no	SMA type	Visit	Treatment	facial nerve	accessory nerve	median nerve	ulnar nerve
1	2	1	-	◇	◇	-11	◇
		2	placebo	◇	◇	+3	-3
		3	-	◇	◇	+4	-2
		4	np	np	np	np	np
2	2	1	-	◆	◆	◆	◆
		2	pyridostigmine	+8	◇	◇	◇
		3	-	+11	◇	◇	◇
		4	placebo	+4	◇	◇	◇
3	3a	1	-	+6	◇	◆	-3
		2	placebo	+10	◇	◇	0
		3	-	+2	◇	◆	◆
		4	pyridostigmine	◆	◆	◆	-11
4	2	1	-	◇	◇	◇	◇
		2	pyridostigmine	+2	◇	-15	◆
		3	-	◇	◇	◇	◇
		4	placebo	-4	◇	◇	-34

5	3a	1	-	◇	◇	◇	◇
		2	placebo	◆	-5	+23	+14
		3	-	+14	-26	◆	-6
		4	pyridostigmine	+28	-11	-2	-3
6	3a	1	-	+8	-23	-6	-4
		2	placebo	0	-28	-12	-8
		3	-	+4	◆	◆	-9
		4	pyridostigmine	+1	-33	◆	-11
7	3b	1	-	◆	◇	◇	◆
		2	placebo	-9	◇	◇	-4
		3	-	◆	◇	◇	-3
		4	pyridostigmine	-6	◇	◇	+8
8	2	1	-	0	◇	-14	◆
		2	pyridostigmine	+1	-14	+2	◆
		3	-	◆	◇	0	◇
		4	placebo	0	-3	-9	-2
9	4	1	-	0	-7	-13	+2
		2	placebo	+1	-1	◆	-1
		3	-	+1	-3	◆	+3
		4	pyridostigmine	+1	+4	+3	+4
10	2	1	-	-9	◇	◇	+2
		2	placebo	-10	+4	◇	◆
		3	-	◆	◇	◇	-2
		4	pyridostigmine	+1	◇	◇	-6
11	3a	1	-	+2	-8	-4	-4
		2	placebo	-3	-3	-10	-9
		3	-	-6	-20	-20	0
		4	np	np	np	np	np

12	3b	1	-	+3	0	-6	+5
		2	placebo	+3	-2	-10	+2
		3	-	+4	-4	-16	-3
		4	pyridostigmine	+3	-10	-6	+3
13	3a	1	-	◇	◇	◇	◇
		2	pyridostigmine	0	◇	-6	+5
		3	-	+1	◇	◆	+1
		4	placebo	+2	◇	◆	+1
14	2	1	-	◇	◇	◇	◆
		2	placebo	◇	◇	◇	◆
		3	-	+7	◇	-11	-15
		4	pyridostigmine	◆	◆	-27	-5
15	2	1	-	◇	◇	◇	◇
		2	pyridostigmine	+5	◇	◇	◇
		3	-	+2	◇	◇	◇
		4	placebo	◆	◇	◇	◇
16	3b	1	-	+2	-13	-6	-6
		2	pyridostigmine	0	-3	-4	+2
		3	-	+1	◆	◇	-3
		4	placebo	+1	0	◆	-3
17	3a	1	-	◆	◆	-7	-2
		2	placebo	0	-8	+9	-1
		3	-	+2	-1	-12	+2
		4		np	np	np	np
18	2	1	-	-1	-18	-27	-8
		2	pyridostigmine	0	-17	-9	-3
		3	-	-4	-14	-4	◆
		4	placebo	0	-16	-12	◆

19	3a	1	-	◇	◇	◇	-2
		2	placebo	0	+7	◆	+3
		3	-	0	-1	-1	+13
		4	pyridostigmine	+1	-8	-12	0
20	3a	1	-	-1	-17	◆	-11
		2	pyridostigmine	+1	-10	-33	◆
		3	-	-3	-17	-38	0
		4	placebo	-2	-7	-27	-7
21	3a	1	-	-3	◆	+9	+3
		2	placebo	+1	◆	+14	-8
		3	-	-6	◇	◇	+2
		4	pyridostigmine	+3	◇	◆	+5
22	3b	1	-	-1	◇	-8	-7
		2	placebo	◇	◇	◇	◇
		3	-	0	◇	+20	-7
		4	pyridostigmine	-2	◇	+1	-5
23	2	1	-	-5	◇	◆	+4
		2	pyridostigmine	-1	◇	◆	-13
		3	-	+8	◆	◇	+1
		4	placebo	-2	◇	◇	-2
24	3b	1	-	+1	-13	◇	-3
		2	pyridostigmine	+1	-14	◇	-3
		3	-	+1	-14	+4	◆
		4	placebo	+1	-13	◇	-3
25	2	1	-	◆	◆	-5	-23
		2	pyridostigmine	+7	◇	◇	-14
		3	-	+2	◇	◇	-15
		4	placebo	+2	◇	◇	-8

26	3a	1	-	-2	-8	◆	◆
		2	pyridostigmine	◆	-4	-10	+1
		3	-	+5	-4	+15	◆
		4	placebo	+2	-7	◆	◆
27	2	1	-	◆	◇	-34	-11
		2	pyridostigmine	+3	◇	◆	-5
		3	-	◆	◆	◆	-39
		4	placebo	+1	◇	◇	◇
28	2	1	-	◇	◇	◇	◇
		2	pyridostigmine	-11	◇	◇	+36
		3	-	+3	◇	◆	-17
		4	placebo	-6	◇	◇	-2
29	3a	1	-	◆	◇	+14	+4
		2	placebo	-2	◇	◇	◆
		3	-	+17	◇	◇	+8
		4	pyridostigmine	+8	◇	◇	-2
30	2	1	-	◆	◇	◆	+11
		2	pyridostigmine	◆	-15	◆	-2
		3	-	-2	◇	◇	+7
		4	placebo	-4	◆	◇	+3
31	2	1	-	-5	◇	◇	+3
		2	placebo	0	◇	◆	-2
		3	-	+2	◇	◆	+1
		4	pyridostigmine	-3	◇	-16	+1
32	3a	1	-	-2	-11	-10	-4
		2	pyridostigmine	◇	◇	◇	◇
		3	-	◆	-21	◆	-3
		4	placebo	-1	-19	-2	-5

33	3a	1	-	-9	◇	◇	+4
		2	pyridostigmine	-1	◇	◇	+20
		3	-	◇	◇	◇	◇
		4	placebo	◆	◆	◆	◆
34	3a	1	-	◇	◇	◇	◇
		2	placebo	+3	-3	-8	-13
		3	-	-2	-1	-22	-2
		4	pyridostigmine	◆	0	◇	-1
35	2	1	-	+2	◇	◇	◇
		2	placebo	+3	◇	◇	0
		3	-	◇	◇	◇	◇
		4	pyridostigmine	◇	◇	◇	◇

◇= *missing due to technical issue or patient discomfort*

◆= missing due to inability to analyze decrement because first CMAP <1

np= not performed due to study discontinuation

[1] Wadman RI, Vrancken AFJE, van den Berg LH, et al. Dysfunction of the neuromuscular junction in spinal muscular atrophy types 2 and 3. Neurology 2012;79(20):2050-5

Supplementary File 1. Pyridostigmine side-effects, extracted from Summary of Product Characteristics (SmPC)

Source: *https://www.medicines.org.uk/emc/product/962/smpc, consulted on July 18, 2021*

4.8 Undesirable effects

- As with all cholinergic products, Mestinon may have unwanted functional effects on the autonomic nervous system. Muscarine-like adverse effects may be exhibited as nausea, vomiting, diarrhoea, abdominal cramps, increased peristaltic and increased bronchial secretion, salivation, bradycardia and miosis.
- The primary nicotinic effects are muscle spasms, fasciculation and muscular weakness.
- Adverse reactions are listed below according to system organ class and frequency. Frequencies are defined according to the following convention:
- Very common (≥1/10), Common (≥1/100 to <1/10), Uncommon (≥1/1,000 to <1/100), Rare (≥1/10,000 to <1/1,000) Very rare (<1/10,000) Not known (cannot be estimated from the available data)

Eye disorders

- Frequency not known: Miosis, increased lacrimation, accommodation disorders

Cardiac disorders

- Frequency not known: Arrhythmia (including bradycardia, tachycardia, AV block), as well as syncope and hypotension (see section 4.9)

Respiratory, thoracic and mediastinal disorders

- Frequency not known: Increased bronchial secretion combined with bronchoconstriction

Gastrointestinal disorders

- Frequency not known: Nausea, vomiting, diarrhoea, abdominal cramps, gastrointestinal hypermotility, salivary hypersecretion

Skin and subcutaneous tissue disorders

- Frequency not known: Rash (disappears usually soon after ceasing of medication. Bromide containing medicines should no longer be used.) Hyperhydrosis

Musculoskeletal and connective tissue disorders

- Frequency not known: Increased muscle weakness fasciculation, tremors and muscle cramps or muscle hypotonia (see section 4.9)

Renal and urinary disorders

- Frequency not known: Urinary urgency
